# Trade-Offs among Sensing, Reporting, and Transmission in Cooperative CRNs

**DOI:** 10.3390/s22134753

**Published:** 2022-06-23

**Authors:** Xiaoying Liu, Kechen Zheng

**Affiliations:** School of Computer Science and Technology, Zhejiang University of Technology, Hangzhou 310023, China; xiaoyingliu@zjut.edu.cn

**Keywords:** cooperative spectrum sensing, cognitive radio networks, reporting period, time trade-off

## Abstract

Cooperative spectrum sensing (CSS) has been verified as an effective approach to improve the sensing performances of cognitive radio networks (CRNs). Compared with existing works that commonly consider fusion with fixed inputs and neglect the duration of the reporting period in the design, we novelly investigate a fundamental trade-off among three periods of CSS: sensing, reporting, and transmission periods, and evaluate the impact of the fusion rule with a varying number of local sensing results. To be specific, the sensing time could be traded for additional mini-slots to report more local sensing results for fusion, or it could be traded for longer transmission time. In the CRNs with a given durations of sensing/reporting/transmission periods, we, respectively, formulate the throughput and collision probability and optimize the throughput under the collision constraint. The theoretical results show that, in the specific value intervals of the sensing parameters, the collision constraint provides an upper bound of the number of mini-slots in the reporting period or a lower bound of the sensing duration. We provide the approach to the maximum throughput in some cases.Finally, numerical results are presented to validate theoretical results.

## 1. Introduction

The fast development of wireless communication technologies and the growing number of high-speed wireless devices are expected to create an increasing demand for spectrum resources [1]. This motivates the advent of cognitive radio to tackle the spectrum scarcity problem by allowing opportunistic spectrum access. In cognitive radio networks (CRNs), secondary users (SUs) are allowed to dynamically access the licensed spectrum allocated to primary users (PUs) when the licensed spectrum is temporally available.

To explore the underutilized spectrum resources, SUs need to adopt fast and effective spectrum-sensing techniques to determine spectrum states. Based on the classification of spectrum-sensing approaches in [2], there are a number of works that focused on spectrum-sensing algorithms [3,4,5,6,7]. Liang et al. [3] designed an energy detection sensing scheme to tackle the sensing–the throughput trade-off of the secondary network. In wideband scenarios under noise uncertainty, Dikmese et al. [4] proposed a cyclic prefix auto-correlation-based spectrum sensing system. By exploiting the signal sparsity, El-Alfi et al. [5] proposed a sub-Nyquist cyclo-stationary detection of GFDM. Sedighi et al. [6] studied constant false-alarm-rate eigenvalue-based detectors for multi-antenna spectrum sensing. Tavana et al. [7] considered the energy and correlation of received signals to suppress error detection. Among these spectrum-sensing techniques, energy detection is the most popular spectrum-sensing technique due to its adequate performance, simple practical realization, and low computational complexity.

Based on the energy-detection technique, many efforts have been geared mainly towards non-cooperative spectrum sensing and cooperative spectrum sensing (CSS). Compared with non-cooperative users, cooperation among users has potential benefits for key performances of CRNs, such as the throughput [8,9], connectivity [10], energy efficiency [11], delay [12], and security [13,14]. Since the unreliability in a single SU’s sensing result reduces the accuracy to a certain extent, CSS has been incorporated to overcome the hidden terminal problem and improve the sensing performance, especially in shadowing/multi-path fading environments [15,16]. In CSS, each user first senses the PU independently and then sends its local sensing result to a fusion center (FC). Then, the FC makes a final decision on the spectrum state by combining all the receiving local sensing results [17,18]. The probability of a false alarm and the probability of detection are two important indicators of spectrum-sensing accuracy. The low probability of a false alarm improves the spectrum-access efficiency, while the high probability of detection reduces the interference with the PU [19]. By exploiting the spatial diversity, cooperative SUs obtain better sensing accuracy by CSS than a single SU.

Incorporating various techniques, such as sensing algorithms [3,4,5,6,7], access strategies, fusion rules, and user cooperation, a number of works [16,20,21,22,23,24,25,26,27,28,29,30,31,32,33,34] have focused on the performance improvements of CRNs with CSS. From the perspective of access strategy, Lee et al. [20] proposed an adaptive CSS scheme using random access. Wang et al. [21,22] proposed a principal–agent-based joint spectrum-sensing and -access framework to thwart the malicious behaviors. Alhamad et al. [16] designed a reporting channel scheme based on random access protocols, and Gharib et al. [23,24] proposed multi-band multi-user CSS schemes for opportunistic spectrum access. From the perspective of fusion rule, Liu et al. [25] maximized the spectral efficiency based on the logical OR rule. Yin et al. [26] maximized the secondary the throughput by the optimization of the fusion rule. Ejaz et al. [27] presented a comparison of hard and soft, combining CSS schemes in heterogeneous CRNs. Golvaei et al. [28] proposed a soft decision algorithm to improve CSS performance for hidden PUs in fading and shadowing environments, which showed better performance than the hard decision algorithm. Yuan et al. [29] and Zhang et al. [30], respectively, proposed a secure fusion strategy to defend against malicious users for CSS. Taking the location impact of different SUs into account, Liu et al. [31] proposed a probability-based fusion rule. Based on the soft rule, Perez et al. [32] presented a new algorithm that jointly estimates the instantaneous SNRs and detects the presence of primary signals. From the perspective of coalition formation, Wang et al. [33] considered overlapping coalition formations for distributed cooperative sensing, where SUs form overlapping coalitions to improve sensing accuracy. Then Jiang et al. [34] formulated a classical coalition formation game model for the throughput maximization and cooperation in spectrum sensing.

Among the aforementioned works, [3,26,34,35] study the throughput maximization of SUs with respect to the fusion rule or sensing parameters such as the detection threshold. However, all four works neglect the duration of the reporting period in the slot structure, and [26,35] do not consider the sensing cooperation among SUs. Moreover, we notice that the duration of the reporting period has been analyzed in terms of sensing performance [16,36], energy consumption [36], and channel utilization [37], respectively. In this regard, the duration of the reporting period has a vital impact on the performances of CRNs, while the throughput and sensing performances of the cooperative CRNs with the design of the reporting period have seldom been studied.

Our work differs from previous works from two perspectives. First, from the perspective of a time trade-off, the duration of the sensing period, the duration of the transmission period, and the number of mini-slots in the reporting period, are, respectively, considered as variables in the formulation and optimization of the secondary the throughput. Second, from the perspective of fusion, our work studies the impact of the fusion rule on the throughput and sensing performances of the cooperative CRNs with a varying number of local sensing results for fusion, which has seldom been studied.

To explore the crucial impact of the reporting period on the throughput and sensing performances, this paper studies the throughput maximization of SUs under the collision constraint in cooperative CRNs, where the CSS process consists of three periods: sensing, reporting, and transmission. Autonomous SUs perform spectrum sensing during the sensing period and then send local sensing results to the FC by a reporting channel. The FC makes a final decision about the licensed spectrum using the “*k*-out-of-*n*” fusion rule during the reporting period. Obviously, there exists a fundamental time trade-off among the sensing period, reporting period, and transmission period, which results in the sensing–the throughput trade-off in the cooperative CRNs. By tackling the time trade-off, the secondary the throughput is formulated and optimized under the collision constraint, which reflects the accuracy of spectrum sensing. By tackling the sensing–the throughput trade-off, we evaluate the impact of the fusion rule with a varying number of local sensing results on the throughput maximization. Then, we summarize the main contributions as follows.

- We formulate the secondary the throughput and collision probability in three cases of the cooperative CRNs, where each time slot in the CSS process consists of the sensing period, reporting period, and transmission period. In the time trade-off, the sensing time could be traded for additional mini-slots to obtain more local sensing results, and it could also be traded for longer transmission times. The impact of the fusion rule with a varying number of local sensing results is studied in the throughput and collision analysis.
- In three cases of the cooperative CRNs, according to the mathematical relationship between *k* and *n*, we, respectively, present a monotonicity analysis of the throughput and collision probability and provide an approach to the maximum the throughput in some cases of the sensing and fusion parameters under the collision constraint.
- The numerical results show that the throughput and the collision probability possess the monotonic property in some value intervals of the sensing and fusion parameters, which is of prime significance for the design of three periods in the slot structure of the CSS process. Moreover, the numerical results demonstrate that, with a given sensing period, the maximal throughput is achieved when the trade-off between the cooperative sensing accuracy, which results from the number of SUs participating in CSS, and transmission time is optimal. With a given reporting period, the maximal throughput is achieved when the trade-off between the local sensing accuracy and transmission time is optimal. With a given transmission period, the maximal throughput is achieved when the cooperative sensing accuracy, which is jointly determined by the local sensing accuracy and the number of SUs participating in CSS, is optimal.

The remainder of this paper is organized as follows. The network model is introduced in Section 2. The the throughput and collision performances of the cooperative CRNs with a given sensing period, reporting period, and transmission period are, respectively, investigated in Section 3, Section 4 and Section 5. The numerical results are given in Section 6, and, finally, this paper is concluded in Section 7.

## 2. Network Model and Notations

In this section, we introduce the cooperative CRNs from three aspects: the network model, the spectrum-sensing model, and the reporting model. We first specify the network topology and slot structure. Then, we specify how SUs cooperatively and opportunistically access the licensed spectrum.

### 2.1. Network Model

**Figure 1:**
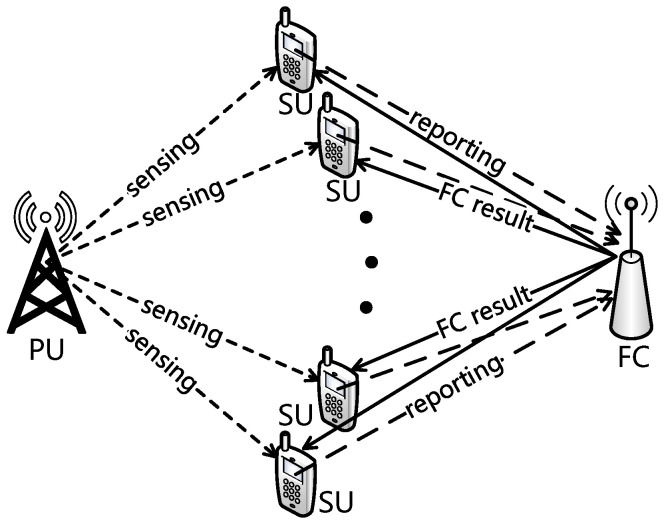
Network model.

**Figure 2:**
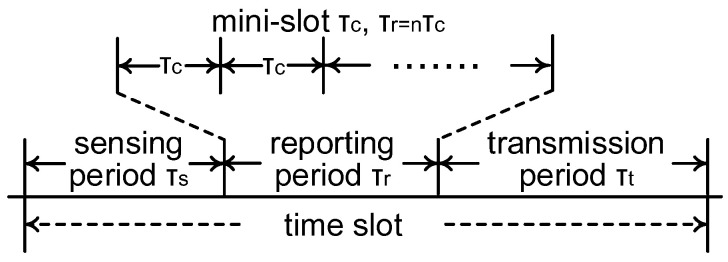
Slot structure.

We consider the CRN scenario where one pair of PUs, one FC, and *N* pairs of SUs coexist. PUs have priorities over SUs to access the licensed spectrum and perform primary packet transmission (the primary receiver is neglected in Figure 1). Spectrum sensing by SUs is essential to avoid excessive interference from SUs to PUs, and the collision probability, which will be defined in the next section, reflects the accuracy of spectrum sensing to a certain extent. When the licensed spectrum is determined is free by the FC, one of the SUs exploits the spectrum and transmits secondary packets to the corresponding secondary receiver (neglected in Figure 1). As shown in Figure 1 and Figure 2, we consider three periods in the slot structure of the cooperative CRNs: a sensing period with duration $\tau_{s}$, a reporting period with duration $\tau_{r}$, and a transmission period with duration $\tau_{t}$. As pointed out by [38], if the SUs have the perfect knowledge of the PU’s communication mechanism, the SUs can be synchronous with the PU’s time slots. Here, we consider that all the SUs and PUs adopt a synchronous slotted protocol with normalized length, where $\tau_{s}+\tau_{r}+\tau_{t}=1$ holds for each time slot by normalization.

During the sensing period, *N* pairs of SUs independently perform spectrum sensing to detect the primary signal in the licensed spectrum by energy detection, which has relatively low computational and implementation complexities [39]. The details of spectrum sensing will be introduced in Section 2.2. Due to the arrival of primary packets following a time-homogeneous random process, the licensed spectrum randomly switches between the free state and the occupied state at each time slot. We let $\pi_{o}$ denote the probability that the licensed spectrum is occupied, and $\pi_{f}$ denote the probability that the licensed spectrum is free, where $\pi_{o}+\pi_{f}=1$.

During the reporting period, the SUs send local sensing results to the FC; the details of the reporting model will be introduced in Section 2.3. By applying a specific fusion rule, the FC makes a final decision about the spectrum state based on the received local sensing results. Then, the FC broadcasts a message containing the final decision about the spectrum state and the identification of a specified SU to SUs [16]. When the FC determines the licensed spectrum to be occupied, the specified SU for the transmission does not exist and would not be contained in the broadcast message. Due to the limited number of bits in the broadcast message, the time duration of the broadcasting period is neglected in the time slot structure.

During the transmission period, if the received broadcast message indicates that the licensed spectrum is decided is free, the randomly specified SU transmits packets to the corresponding secondary receiver (neglected in Figure 1). Otherwise, the secondary transmission does not occur during the transmission period. To evaluate the secondary the throughput of the CRNs from the perspective of the actual transmission time, we define the throughput as the average time duration of the secondary transmission that does not collide with the primary transmission in the normalized time slot, and this definition is similar to that of the average normalized the throughput in [25].

(1) $$\lambda =\lim_{T\to \infty }\frac{\sum_{i=1}^{T}\tau_{ti}}{T},$$

where $\tau_{ti}$ denotes the duration of the secondary packet transmission that does not collide with the primary packet transmission at slot *i*, *T* represents the observed number of time slots, and 1≤i≤T holds. If the FC correctly determines the licensed spectrum is free at slot *i*, the secondary packet transmission occurs during the transmission period, and $\tau_{ti}=\tau_{t}$ holds. Otherwise, $\tau_{ti}=0$ holds at slot *i*. Therefore, the average normalized the throughput λ in (1) represents the ratio of the secondary packet transmission duration without colliding with the primary packet transmission to one time slot from a long-term perspective. According to the slot structure in Figure 2, the sensing period and the number of mini-slots in the reporting period have great impacts on the throughput.

### 2.2. Spectrum-Sensing Model

Various spectrum-sensing techniques have been proposed to determine the spectrum state, including energy detection [40], matched-filter detection [41], eigenvalue-based detection [42], and cyclostationarity-based detection [43]. Among these four techniques, we employ energy detection to detect the spectrum in the sensing period due to its adequate performance, simple practical realization, and low computational complexity [9,39]. During the sensing period, local sensing is implemented with the energy detector in each SU. The energy detector in the SU conducts spectrum sensing by executing a binary hypothesis test. According to [3], the binary hypothesis test for a given SU is expressed as

$$\left\{\begin{array}{cc} H_{0}: & y(m)=l(m),\\ H_{1}: & y(m)=s(m)+l(m), \end{array}\right.$$

where y(m) represents the *m*-th sample of a SU’s energy detector, and *m* is a positive integer. s(m) and l(m) represent the PU’s signal and noise, respectively, and they are modeled as independent circularly symmetric complex Gaussian (CSCG) random processes with variances $\sigma_{p}^{2}$ and $\sigma_{n}^{2}$, respectively [3]. Let r∈{0(free),1(occupied)} denote the sensing result. Let γ denote the ratio of $\sigma_{p}^{2}$ to $\sigma_{n}^{2}$, $f_{s}$ denote the sampling frequency, and ϵ denote the detection threshold. According to [3], under hypothesis $H_{0}$, the probability of a false alarm, denoted by $p_{f}\left(\tau_{s}\right)$, can be approximately expressed as

(2) $$p_{f}\left(\tau_{s}\right)=P\left(r=1|H_{0}\right)=Q\left(\left(\frac{\epsilon }{\sigma_{n}^{2}}-1\right)\sqrt{\tau_{s}f_{s}}\right),$$

where $Q\left(x\right)=\frac{1}{\sqrt{2\pi }}\int_{x}^{\infty }\exp\left(-\frac{u^{2}}{2}\right)du$ is the complementary distribution function of the standard Gaussian distribution. The probability of a false alarm represents the probability that the sensing result is occupied while the actual spectrum state is free. Under hypothesis $H_{1}$, the probability of detection, denoted by $p_{d}\left(\tau_{s}\right)$, can be approximately expressed as

(3) $$p_{d}\left(\tau_{s}\right)=P\left(r=1|H_{1}\right)=Q\left(\left(\frac{\epsilon }{\sigma_{n}^{2}}-\gamma -1\right)\sqrt{\frac{\tau_{s}f_{s}}{2\gamma +1}}\right).$$

The probability of detection represents the probability that the sensing result is occupied while the actual spectrum state is occupied. Each SU is assumed to share the same sampling frequency, signal-to-noise ratio, and detection threshold for local sensing results by energy detection as in [16,26]; thus, the identical probability of detection $p_{d}\left(\tau_{s}\right)$ and that of a false alarm $p_{f}\left(\tau_{s}\right)$ for each SU could be adopted. Based on (2) and (3), we let $p_{f}\left(\tau_{s}\right)=Q\left(c_{1}\sqrt{\tau_{s}}\right)$ and $p_{d}\left(\tau_{s}\right)=Q\left(c_{2}\sqrt{\tau_{s}}\right)$ for simplicity and have

(4) $$c_{1}=\left(\frac{\epsilon }{\sigma_{n}^{2}}-1\right)\sqrt{f_{s}},$$

and

(5) $$c_{2}=\left(\frac{\epsilon }{\sigma_{n}^{2}}-\gamma -1\right)\sqrt{\frac{f_{s}}{2\gamma +1}}.$$

With respect to the definitions of detection probability $p_{d}\left(\tau_{s}\right)$ in (3) and false alarm probability $p_{f}\left(\tau_{s}\right)$ in (2), the cases where $p_{d}\left(\tau_{s}\right)>0.5$ and $p_{f}\left(\tau_{s}\right)<0.5$ are considered throughout this paper. Otherwise, the probability of correctly determining the spectrum state would be less than a half, and it is meaningless for SUs to perform spectrum sensing with such low accuracy. Based on the formula of Q(x) and the aforementioned value intervals of $p_{d}\left(\tau_{s}\right)$ and $p_{f}\left(\tau_{s}\right)$, we could easily obtain that $c_{1}>0$ in (4) and $c_{2}<0$ in (5).

### 2.3. Reporting Model

In the reporting model, we consider that the reporting period consists of *n* mini-slots with equal duration $\tau_{c}$. Actually, $\tau_{c}$ denotes the reporting duration for one SU. The number of mini-slots *n* is considered to be a positive variable in the time trade-off of three periods for the throughput optimization, where *n* is a positive integer, $\tau_{r}=n\tau_{c}$, and 1≤n≤N holds. We adopt random access in the reporting period, where the slotted Aloha (S-Aloha) is adopted due to its easy implementation and low complexity [16]. In the S-Aloha protocol, each SU randomly selects one of the *n* mini-slots to transmit its local sensing result to the FC [16]. Based on the capture effect, we notice that, in the presence of other overlapping or interfering packets, the strongest packet could capture the receiver when the power strength of the strongest packet is at least *d* times the power strength of the second-strongest packet, where d=1 holds for a perfect capture [16,44]. Throughout this paper, we consider the case that the FC receives local sensing results by S-Aloha protocol with perfect capture. Namely, the FC could successfully receive a local sensing result from a non-repeatable SU at each mini-slot.

Without loss of generality, we adopt the “*k*-out-of-*n*” fusion rule to make a final decision about the spectrum state. There are *n* SUs participating in the CSS, and the local sensing result is independently made by each SU. Then, the FC decides the licensed spectrum is free if there are *k* or more local sensing results being idle. The closed interval of *k* is [1,n], and *k* is a positive integer. Since the local sensing results obtained by the SUs are independent of each other for CSS, we denote $P_{f}\left(n,\tau_{s}\right)$ as the overall false-alarm probability and denote $P_{d}\left(n,\tau_{s}\right)$ as the overall detection probability. According to [9,26], $P_{f}\left(n,\tau_{s}\right)$ and $P_{d}\left(n,\tau_{s}\right)$ are, respectively, given by

(6) Pf(n,τs)=∑x=0k−1nx1−pf(τs)xpf(τs)n−x,

and

(7) Pd(n,τs)=∑x=0k−1nx1−pd(τs)xpd(τs)n−x,

where nx=n!x!(n−x)! holds. The “*k*-out-of-*n*” fusion rule is reduced to the “Logic-AND” rule when k=1, and it is reduced to the “Logic-OR” rule when k=n [3].

## 3. Performance Analysis of the CRN with Given Sensing Period

In this section, we tackle the trade-off between the duration of the reporting period and that of the transmission period for the throughput optimization. Namely, the number of mini-slots *n* in the reporting period could be traded for the transmission time. We start our analysis by formulating the throughput and collision probability and maximizing the secondary the throughput of the cooperative CRN under the collision constraint.

Based on the definition of the throughput in (1), the throughput of the CRN with a given sensing period $\tau_{s}$, denoted by $\lambda_{s}$, is given by

(8) λs=πf(1−τs−τr)(1−Pf(n,τs))=πf(1−τs−nτc)∑x=knnx1−pfxpfn−x=πf(1−τs−nτc)(1−∑x=0k−1nx1−pfxpfn−x),

where $p_{f}\left(\tau_{s}\right)$ in (2) is simplified as $p_{f}$ in (8), since $\tau_{s}$ is given in this section. Then we introduce the collision probability. A collision occurs when the FC determines that the licensed spectrum is free while the licensed spectrum is actually occupied. Hence, the collision probability represents the probability that the SU accesses the occupied spectrum and collides with the primary signal.

(9) Ps=πo(1−Pd(n,τs))=πo∑x=knnx1−pdxpdn−x=πo(1−∑x=0k−1nx1−pdxpdn−x),

where $p_{d}\left(\tau_{s}\right)$ in (3) is simplified as $p_{d}$, since $\tau_{s}$ is given in this section. To protect the primary transmission from the access of SUs to some extent, SUs must maintain the collision constraint such that the collision probability remains below the maximum permissible collision probability $P_{c}$, which is commonly viewed as a pre-designed value.

### 3.1. Throughput Analysis

To tackle the trade-off between the duration of the reporting period and that of the transmission period, the number of mini-slots *n* is considered to be a variable to maximize the throughput $\lambda_{s}$. Though *n* is defined as a positive integer, the following analysis of the continuous function $\lambda_{s}$ that regards *n* as a positive continuous variable applies to the throughput analysis of the positive integer *n* in the closed interval [1,N]. Then, we differentiate $\lambda_{s}$ in (8) with respect to *n* as

(10) 1πf∂λs∂n=−τc(1−∑x=0k−1nx1−pfxpfn−x)−(1−τs−nτc)∑x=0k−1∂nx∂n1−pfxpfn−x−(1−τs−nτc)lnpf∑x=0k−1nx1−pfxpfn−x,

where the *k* in the “*k*-out-of-*n*” fusion rule is considered to be independent of *n*; thus, ∂nx∂n is

(11) ∂nx∂n=∂n!x!(n−x)!∂n=∂n!(n−x)!∂nx!=∑i=1x1n−x+i∏j=1x(n−x+j)x!.

Based on Euler’s constant [45], we approximate the numerator on the right-hand side of (11) as

(12) $$\begin{array}{c} \;\sum_{i=1}^{x}\frac{1}{n\;-\;x\;+\;i}\;\approx \;\ln\left(n\;+\;1\right)\;-\;\ln\left(n\;-\;x\;+\;1\right)\;=\;\ln\left(\;\frac{n+1}{n\;-\;x\;+\;1}\;\right). \end{array}$$

By combining (11) with (12), we have

(13) ∂nx∂n≈ln(1+xn−x+1)∏j=1x(n−x+j)x!=lnn+1n−x+1nx.

We approximate the second term on the right-hand side of (10) by substituting (13) into (10) as

(14) −(1−τs−nτc)∑x=0k−1∂nx∂n1−pfxpfn−x≈−(1−τs−nτc)∑x=0k−1lnn+1n−x+1nx1−pfxpfn−x.

We further simplify the right-hand side of (10) by substituting (14) into (10) as

(15) 1πf∂λs∂n=−τc(1−∑x=0k−1nx1−pfxpfn−x)−(1−τs−nτc)∑x=0k−1lnpf(n+1)n−x+1nx1−pfxpfn−x.

We observe from (15) that nx>0, $p_{f}>0$, $1-p_{f}>0$, $\tau_{c}>0$, and $1-\tau_{s}-n\tau_{c}>0$ hold; thus, the first term on the right-hand side of (15) is negative. Only the multiplicator $\ln\left(\frac{p_{f}\left(n+1\right)}{n-x+1}\right)$ in the second term on the right-hand side of (15) needs to be further discussed as

(16) $$\begin{array}{c} \ln\left(\frac{p_{f}\left(n+1\right)}{n-x+1}\right)<0\Rightarrow \frac{p_{f}\left(n+1\right)}{n-x+1}<1\Rightarrow x<\left(1-p_{f}\right)\left(n+1\right). \end{array}$$

With the non-negative integer *x* in the closed interval [0,k−1], we obtain the sufficient condition that the second term on the right-hand side of (15) is positive as

(17) $$k<\left(1-p_{f}\right)\left(n+1\right)+1.$$

When the value interval of *k* in (17) holds, the first term on the right-hand side of (15) is negative, while the second term is positive. Due to the definition of $\tau_{c}$ and the value comparison between $\tau_{c}$ and $\left(1-\tau_{s}-n\tau_{c}\right)$, we deduce that the throughput $\lambda_{s}$ increases with the number of mini-slots *n* when (17) holds. Otherwise, with the increase of *k* and $p_{f}$, the throughput $\lambda_{s}$ turns to decrease with the number of mini-slots *n*. Since the *k* in the “*k*-out-of-*n*” fusion rule is independent of *n*, 1≤k≤n holds. We summarize the observations from (10)–(17) as follows.

- When $k\in \left[\left(1-p_{f}\right)\left(n+1\right)+1,n\right]$, the monotonicity of the throughput $\lambda_{s}$ depends on $p_{f}$ and *k*. On the right-hand side of (15), the first term is negative, the summation of $x\in \left[0,\left(1-p_{f}\right)\left(n+1\right)+1\right)$ in the second term is positive, while the summation of $x\in \left[\left(1-p_{f}\right)\left(n+1\right)+1,k\right]$ in the second term is negative. Compared with the second item as follows, $\lambda_{s}$ is more likely to decrease with the number of mini-slots *n* due to the larger value of *k*.
- When $k\in \left[1,\left(1-p_{f}\right)\left(n+1\right)+1\right)$, the first term on the right-hand side of (15) is negative, while the second term on the right-hand side of (15) is positive. By comparing the first and second terms, we deduce from (15)–(17) that the the throughput $\lambda_{s}$ increases with the number of mini-slots *n*.

We also deduce from the value interval of *k* in the above first item that, when $p_{f}<\frac{2}{n+1}$, the lower bound of *k* in the first item is larger than *n*; thus, the above first item does not exist. With respect to the above value interval of $p_{f}$, the interval of *k* in the second item occupies the majority part. Moreover, with the increase of *k*, it is easy to observe from (15) and (17) that the throughput $\lambda_{s}$ is more likely to increase with *n*.

### 3.2. Collision Analysis

Since the number of mini-slots *n* is considered to be a variable in this section, in order to investigate the monotonicity of the collision probability, we differentiate $P_{s}$ in (9) with respect to *n* as

(18) 1πo∂Ps∂n=−∑x=0k−1∂nx∂n1−pdxpdn−x−lnpd∑x=0k−1nx1−pdxpdn−x.

Based on (13), we simplify (18) as

(19) 1πo∂Ps∂n≈−∑x=0k−1lnn+1n−x+1nx1−pdxpdn−x−lnpd∑x=0k−1nx1−pdxpdn−x=−∑x=0k−1lnpd(n+1)n−x+1nx1−pdxpdn−x.

We observe from (19) that $\pi_{o}>0$, nx>0, $p_{d}>0$, and $1-p_{d}>0$ hold; thus, only the multiplicator $\ln(\frac{p_{d}\left(n+1\right)}{n-x+1})$ in (19) needs to be further discussed for the monotonicity of the collision probability $P_{s}$. Then, we have

(20) $$\begin{array}{c} \ln\left(\frac{p_{d}\left(n+1\right)}{n-x+1}\right)<0\Rightarrow \frac{p_{d}\left(n+1\right)}{n-x+1}<1\Rightarrow x<\left(1-p_{d}\right)\left(n+1\right). \end{array}$$

As *x* is a non-negative integer in the closed interval [0,k−1], we obtain the sufficient condition that the right-hand side of (18) is positive as

(21) $$k<\left(1-p_{d}\right)\left(n+1\right)+1.$$

Based on (18)–(21), we deduce that the collision probability $P_{s}$ increases with the number of mini-slots *n* when (21) holds. Then, we summarize the observations from (18)–(21) as follows.

- When $k\in [\left(1-p_{d}\right)\left(n+1\right)+1,n]$, the right-hand side of (18) depends on $p_{d}$ and *k*. Specifically, in (19), the summation of $x\in \left[0,\left(1-p_{d}\right)\left(n+1\right)+1\right)$ is positive, while the summation of $x\in \left[\left(1-p_{d}\right)\left(n+1\right)+1,k\right]$ is negative.
- When $k\in [1,\left(1-p_{d}\right)\left(n+1\right)+1)$, the collision probability $P_{s}$ increases with the number of mini-slots *n*. Thus, the collision probability $P_{s}$ and the maximum permissible collision probability $P_{c}$ provide an upper bound of *n* for the throughput optimization.

Moreover, with the increase of $p_{d}$ or *k*, we deduce that the collision probability $P_{s}$ is more likely to decrease with the number of mini-slots *n* based on (19).

### 3.3. Throughput Optimization

Based on the monotonicity analysis of $\lambda_{s}$ and $P_{s}$ introduced in Section 3.1 and Section 3.2, we formulate the throughput optimization problem as

(22) $$\begin{array}{cc} \; & \max_{n}\;\lambda_{s} \end{array}$$

(23) $$\begin{array}{cc} \; & s.t.\;P_{s}\leq P_{c}. \end{array}$$

Equation (22) represents the goal of maximizing the throughput $\lambda_{s}$ and aims to optimize the time trade-off between the transmission period and the number of mini-slots in the reporting period. Equation (23) represents that the collision probability of the CRN with a given sensing period $P_{s}$ should not exceed the maximum permissible collision probability $P_{c}$, by which the PUs can be sufficiently protected from illegal access by the SUs [9]. The maximum permissible collision probability $P_{c}$ is a pre-designed value, which is often determined by the network designer and is independent of other variables [3,9]. Since the throughput depends on the duration of the sensing/reporting/transmission period, and the collision probability reflects the accuracy of spectrum sensing to a certain extent, we optimize the secondary the throughput under the collision constraint to tackle the sensing–the throughput trade-off in the cooperative CRNs. In other words, with the constraint of the sensing performance that requires the time of local sensing and reporting, we aim to provide more transmission time for higher the throughput. With respect to the value intervals of $p_{f}$ and $p_{d}$ in Section 2.2, we have

(24) $$\begin{array}{c} 1>p_{d}>0.5>p_{f}>0\Rightarrow \left(1-p_{f}\right)\left(n+1\right)>\left(1-p_{d}\right)\left(n+1\right). \end{array}$$

Based on the value interval of *k* in Section 3.1 and Section 3.2, we summarize the throughput optimization under the collision constraint by optimizing the number of mini-slots *n* as follows.

- When $k\in \left[\left(1-p_{f}\right)\left(n+1\right)+1,n\right]$, the monotonicities of the throughput $\lambda_{s}$ and the collision probability $P_{s}$ depend on the values of $p_{f}$, $p_{d}$, and *k*. Given specified values of the aforementioned parameters, the optimal *n* could be determined.
- When $k\in \left[\left(1-p_{d}\right)\left(n\;+1\right)\;+\;1,\left(1-p_{f}\right)\left(n\;+1\right)\;+\;1\right]$, the throughput $\lambda_{s}$ increases with *n*, while the monotonicity of $P_{s}$ depends on the values of $p_{d}$ and *k*.
- When $k\in [1,\left(1-p_{d}\right)\left(n+1\right)+1]$, the collision probability $P_{s}$ increases with the number of mini-slots *n*. Thus, the collision probability $P_{s}$ and the maximum permissible collision probability $P_{c}$ provide an upper bound of *n*. The monotonicity of the throughput $\lambda_{s}$ is similar to the second item; thus, the provided upper bound of *n* achieves the maximum the throughput.

## 4. Performance Analysis of the CRN with a Given Reporting Period

In this section, we tackle the trade-off between the duration of the sensing period $\tau_{s}$ and that of the transmission period $\tau_{t}$ for the throughput optimization. Namely, the sensing time $\tau_{s}$ could be traded for transmission time. We start our analysis by formulating the throughput $\lambda_{r}$ and the collision probability $P_{r}$ and maximizing the secondary the throughput of the cooperative CRN under the collision constraint.

Based on the definition of the throughput in (1), the throughput of the CRN with a given reporting period, denoted by $\lambda_{r}$, is given by

(25) λr=πf(1−τs−nτc)∑x=knnx1−pf(τs)xpf(τs)n−x.

The collision probability of the CRN with a given reporting period, denoted by $P_{r}$, is given by

(26) Pr=πo∑x=knnx1−pd(τs)xpd(τs)n−x.

Given the duration of the reporting period in this section, the number of mini-slots *n* in the reporting period is viewed as given in (25) and (26).

### 4.1. Throughput Analysis

To tackle the trade-off between the duration of the sensing period and that of the transmission period, we optimize the duration of the sensing period $\tau_{s}$ to maximize the throughput $\lambda_{r}$. We differentiate $\lambda_{r}$ in (25) with respect to $\tau_{s}$ as

(27) 1πf∂λr∂τs=−∑x=knnx1−pf(τs)xpf(τs)n−x+(1−τs−nτc)∂pf(τs)∂τs×∑x=knnx1−pf(τs)xpf(τs)n−xn−xpf(τs)−x1−pf(τs),

where *n* and *k* are independent of $\tau_{s}$, and we have the first-order partial derivative of $p_{f}\left(\tau_{s}\right)$ with respect to $\tau_{s}$ as

(28) $$\begin{array}{c} \frac{\partial p_{f}\left(\tau_{s}\right)}{\partial \tau_{s}}=\frac{\partial Q(c_{1}\sqrt{\tau_{s}})}{\partial \tau_{s}}=-\frac{c_{1}}{2\sqrt{2\pi \tau_{s}}}e^{-\frac{c_{1}^{2}\tau_{s}}{2}}. \end{array}$$

Due to $c_{1}>0$, given in Section 2.2, we deduce that $\frac{\partial p_{f}\left(\tau_{s}\right)}{\partial \tau_{s}}$ in (28) is negative. As the duration of the sensing period $\tau_{s}\in \left(0,1-n\tau_{c}\right)$ holds, we have

(29) $$\frac{\partial p_{f}\left(\tau_{s}\right)}{\partial \tau_{s}}{|}_{\tau_{s}\to 0^{+}}=-\infty ,$$

and

(30) $$\frac{\partial p_{f}\left(\tau_{s}\right)}{\partial \tau_{s}}{|}_{\tau_{s}\to {\left(1-n\tau_{c}\right)}^{-}}=-\frac{c_{1}}{2\sqrt{2\pi (1-n\tau_{c})}}e^{-\frac{c_{1}^{2}\left(1-n\tau_{c}\right)}{2}},$$

where $\tau_{s}\to 0^{+}$ represents that $\tau_{s}$ tends to 0 from the right of 0, and $\tau_{s}\to {\left(1-n\tau_{c}\right)}^{-}$ represents that $\tau_{s}$ tends to $\left(1-n\tau_{c}\right)$ from the left of $\left(1-n\tau_{c}\right)$. Then, we discuss the multiplicator $\left(\frac{n-x}{p_{f}\left(\tau_{s}\right)}-\frac{x}{1-p_{f}\left(\tau_{s}\right)}\right)$ in (27) as follows.

(31) $$\begin{array}{cc} \frac{n-x}{p_{f}\left(\tau_{s}\right)}-\frac{x}{1-p_{f}\left(\tau_{s}\right)}<0 & \Rightarrow x>(1-p_{f}\left(\tau_{s}\right))n. \end{array}$$

Since *x* is a positive integer in the closed interval [k,n] based on (25), the sufficient condition that the second term on the right-hand side of (27) is positive can be deduced as

(32) $$k>(1-p_{f}\left(\tau_{s}\right))n.$$

Then, we summarize the observations from (27)–(32) as follows.

- When $k\in (\left(1-p_{f}\left(\tau_{s}\right)\right)n,n]$, the first term on the right-hand side of (27) is negative, while the second term on the right-hand side of (27) is positive. Based on (28)–(30), the throughput $\lambda_{r}$ increases with the duration of the sensing period $\tau_{s}$ when $\tau_{s}$ tends to 0. Otherwise, the throughput $\lambda_{r}$ decreases with $\tau_{s}$.
- When $k\in [1,\left(1-p_{f}\left(\tau_{s}\right)\right)n]$, the right-hand side of (27) depends on the values of $p_{f}\left(\tau_{s}\right)$ and *k*. On the right-hand side of (27), the first term is negative, the second term with $x\in \left[k,(1-p_{f}\left(\tau_{s}\right))n\right]$ is negative, while the second term with $x\in \left((1-p_{f}\left(\tau_{s}\right))n,n\right]$ is positive. Compared with the above first item, the throughput $\lambda_{r}$ is more likely to decrease with $\tau_{s}$ due to the smaller value of *k*.

Note that the Equations (27)–(32) are derived using differential and integral calculus to analyze the monotonocity of the throughput with respect to the sensing duration $\tau_{s}$. They are the intermediate results in our analysis of the trade-off among the three periods in the CSS,= and do not exist in the existing literature. Moreover, based on (28), the second-order partial derivative of $p_{f}\left(\tau_{s}\right)$ with respect to $\tau_{s}$ is

(33) $$\frac{\partial^{2}p_{f}\left(\tau_{s}\right)}{\partial \tau_{s}^{2}}=\frac{c_{1}}{4\sqrt{2\pi \tau_{s}^{3}}}e^{-\frac{c_{1}^{2}\tau_{s}}{2}}+\frac{c_{1}^{3}}{4\sqrt{2\pi \tau_{s}}}e^{-\frac{c_{1}^{2}\tau_{s}}{2}}.$$

Due to $c_{1}>0$, given in Section 2.2, we deduce that the second-order partial derivative $\frac{\partial^{2}p_{f}\left(\tau_{s}\right)}{\partial \tau_{s}^{2}}$ is positive. Comparing the first and second terms on the right-hand side of (27), we infer that the value of $\frac{\partial p_{f}\left(\tau_{s}\right)}{\partial \tau_{s}}$ in (28)–(30) plays a key role in (27). Moreover, we infer from (27) and (32) that, with the increase of *k* (or with the decrease of *n*), the throughput $\lambda_{r}$ is more likely to increase with $\tau_{s}$.

### 4.2. Collision Analysis

To analyze the monotonicity of the collision probability, we differentiate $P_{r}$ with respect to $\tau_{s}$ as

(34) 1πo∂Pr∂τs=∂pd(τs)∂τs∑x=knnx1−pd(τs)xpd(τs)n−xn−xpd(τs)−x1−pd(τs),

where we have

(35) $$\begin{array}{c} \frac{\partial p_{d}\left(\tau_{s}\right)}{\partial \tau_{s}}=\frac{\partial Q(c_{2}\sqrt{\tau_{s}})}{\partial \tau_{s}}=-\frac{c_{2}}{2\sqrt{2\pi \tau_{s}}}e^{-\frac{c_{2}^{2}\tau_{s}}{2}}. \end{array}$$

Due to $c_{2}<0$ in (5), we deduce that the first-order partial derivative $\frac{\partial p_{d}\left(\tau_{s}\right)}{\partial \tau_{s}}$ in (35) is positive.

(36) $$\frac{\partial p_{d}\left(\tau_{s}\right)}{\partial \tau_{s}}{|}_{\tau_{s}\to 0^{+}}=+\infty ,$$

(37) $$\frac{\partial p_{d}\left(\tau_{s}\right)}{\partial \tau_{s}}{|}_{\tau_{s}\to {\left(1\;-\;n\tau_{c}\right)}^{-}}\;=\;-\frac{c_{2}}{2\sqrt{2\pi (1\;-\;n\tau_{c})}}e^{-\frac{c_{2}^{2}\left(1\;-\;n\tau_{c}\right)}{2}},$$

and

(38) $$\frac{\partial^{2}p_{d}\left(\tau_{s}\right)}{\partial \tau_{s}^{2}}=\frac{c_{2}}{4\sqrt{2\pi \tau_{s}^{3}}}e^{-\frac{c_{2}^{2}\tau_{s}}{2}}+\frac{c_{2}^{3}}{4\sqrt{2\pi \tau_{s}}}e^{-\frac{c_{2}^{2}\tau_{s}}{2}}.$$

Similar to the analysis of the collision probability (21), (22) in Section 3.2, since $\pi_{o}>0$, nx>0, $1-p_{d}\left(\tau_{s}\right)>0$, and $p_{d}\left(\tau_{s}\right)>0$ hold in (34), we obtain the sufficient condition that the right-hand side of (34) is negative as

(39) $$\begin{array}{cc} \frac{n-x}{p_{d}\left(\tau_{s}\right)}-\frac{x}{1-p_{d}\left(\tau_{s}\right)}<0 & \Rightarrow x>(1-p_{d}\left(\tau_{s}\right))n. \end{array}$$

Therefore, we summarize the observations from (34)–(39) as follows.

- When $k\in (\left(1-p_{d}\left(\tau_{s}\right)\right)n,n]$, the right-hand side of (34) is negative, and the collision probability $P_{r}$ decreases with $\tau_{s}$. Thus, the maximum permissible collision probability $P_{c}$ provides a lower bound of $\tau_{s}$ for throughput optimization.
- When $k\in [1,\left(1-p_{d}\left(\tau_{s}\right)\right)n]$, the right-hand side of (34) depends on $p_{d}\left(\tau_{s}\right)$ and *k*. On the right-hand side of (34), the summation of $x\in \left[k,(1-p_{d}\left(\tau_{s}\right))n\right]$ is positive, while the summation of $x\in \left((1-p_{d}\left(\tau_{s}\right))n,n\right]$ is negative. Compared with the above first item, the collision probability $P_{r}$ is more likely to increase with $\tau_{s}$, due to the smaller value of *k*.

With respect to the value interval $p_{d}\left(\tau_{s}\right)>0.5$, the value interval of *k* in the first item occupies the majority the interval [1,n]. Therefore, (34) and (39) indicate that, with the increase of $p_{d}\left(\tau_{s}\right)$ or *k*, the collision probability $P_{r}$ is more likely to decrease with $\tau_{s}$.

### 4.3. Throughput Optimization

Based on the monotonicity analysis in Section 4.1 and Section 4.2, we formulate the throughput optimization problem as

(40) $$\begin{array}{cc} \; & \max_{\tau_{s}}\;\lambda_{r} \end{array}$$

(41) $$\begin{array}{cc} \; & s.t.\;P_{r}\leq P_{c}. \end{array}$$

The variable of the throughput optimization in (40) is the duration of the sensing period $\tau_{s}$. Equation (40) aims to optimize the time trade-off between the sensing period and the transmission period. With respect to the value intervals of $p_{f}\left(\tau_{s}\right)$ and $p_{d}\left(\tau_{s}\right)$ in Section 2.2, we have

(42) $$\begin{array}{c} 1>p_{d}\left(\tau_{s}\right)>0.5>p_{f}\left(\tau_{s}\right)>0\Rightarrow n\left(1-p_{f}\left(\tau_{s}\right)\right)>n\left(1-p_{d}\left(\tau_{s}\right)\right). \end{array}$$

Based on the value interval of *k* discussed in Section 4.1 and Section 4.2, we summarize the throughput optimization as follows

- When $k\in (\left(1-p_{f}\left(\tau_{s}\right)\right)n,n]$, the collision probability $P_{r}$ decreases with $\tau_{s}$; thus, the maximum permissible collision probability $P_{c}$ provides a lower bound of $\tau_{s}$ in the interval $\left(0,1-n\tau_{c}\right]$. As $\frac{\partial \lambda_{r}}{\partial \tau_{s}}$ turns from positive to negative with the increase of $\tau_{s}$ in $\left(0,1-n\tau_{c}\right)$, the optimal $\tau_{s}$ depends on the lower bound of $\tau_{s}$ and the value of $\tau_{s}$ that satisfies $\frac{\partial \lambda_{r}}{\partial \tau_{s}}=0$.
- When $k\in (\left(1-p_{d}\left(\tau_{s}\right)\right)n,\left(1-p_{f}\left(\tau_{s}\right)\right)n]$, the collision probability $P_{r}$ decreases with $\tau_{s}$, and a lower bound of $\tau_{s}$ is also provided. The optimal $\tau_{s}$ depends on the values of $c_{2}$ and *k*.
- When $k\in [1,\left(1-p_{d}\left(\tau_{s}\right)\right)\left(n+1\right)]$, the monotonicities of the throughput $\lambda_{r}$ and collision probability $P_{r}$ depend on the values of $c_{1}$, $c_{2}$, and *k*.

### 4.4. Special Case

In this subsection, we analyze the throughput and collision performances of the CRN with n=1; thus, k=1 holds due to the definition of the “*k*-out-of-*n*” fusion rule in Section 2.3. The case n=1 corresponds to the CRN scenario with only one pair of SUs. Based on the throughput $\lambda_{r}$ in (25) and the collision probability $P_{r}$ in (26), we formulate the optimization problem by specifying n=1 and k=1 as

(43) $$\begin{array}{cc} \max\; & \lambda_{r}{|}_{n=1}=\pi_{f}\left(1-\tau_{s}-n\tau_{c}\right)\left(1-p_{f}\left(\tau_{s}\right)\right) \end{array}$$

(44) $$\begin{array}{cc} s.t.\; & P_{r}{|}_{n=1}=\pi_{o}\left(1-p_{d}\left(\tau_{s}\right)\right)\leq P_{c}. \end{array}$$

Based on (35), it is easy to deduce that $P_{r}{|}_{n=1}$ decreases with $\tau_{s}$; thus, the collision constraint (44) provides a lower bound of $\tau_{s}$ for the optimization problem. The lower bound of $\tau_{s}$ is given by

(45) $$\tau_{s}\geq {\left(\frac{Q^{-1}\left(1-\frac{P_{c}}{\pi_{o}}\right)}{c_{2}}\right)}^{2}.$$

Then, we analyze the monotonicity of $\lambda_{r}{|}_{n=1}$ and differentiate $\lambda_{r}{|}_{n=1}$ with respect to $\tau_{s}$ as

(46) $$\frac{1}{\pi_{f}}\frac{\partial \lambda_{r}{|}_{n=1}}{\partial \tau_{s}}=-1+p_{f}\left(\tau_{s}\right)-\left(1-\tau_{s}-n\tau_{c}\right)\frac{\partial p_{f}\left(\tau_{s}\right)}{\partial \tau_{s}}.$$

We again differentiate $\frac{\partial \lambda_{r}{|}_{n=1}}{\partial \tau_{s}}$ with respect to $\tau_{s}$ as

(47) $$\frac{1}{\pi_{f}}\frac{\partial^{2}\lambda_{r}{|}_{n=1}}{\partial \tau_{s}^{2}}=\frac{2\partial p_{f}\left(\tau_{s}\right)}{\partial \tau_{s}}-\left(1-\tau_{s}-n\tau_{c}\right)\frac{\partial^{2}p_{f}\left(\tau_{s}\right)}{\partial \tau_{s}^{2}}.$$

Based on (28) and (33), we deduce that the right-hand side of (47) is negative. Moreover, we have

(48) $$\frac{1}{\pi_{f}}\frac{\partial \lambda_{r}{|}_{n=1}}{\partial \tau_{s}}{|}_{\tau_{s}\to 0^{+}}>0,$$

and

(49) $$\frac{1}{\pi_{f}}\frac{\partial \lambda_{r}{|}_{n=1}}{\partial \tau_{s}}{|}_{\tau_{s}\to {\left(1-n\tau_{c}\right)}^{-}}<0.$$

Based on (46)–(49), we deduce that there exists a unique $\tau_{s}^{*}\in \left(0,1-n\tau_{c}\right)$ that satisfies the first-order partial derivative of $\lambda_{r}{|}_{n=1}$ with respect to $\tau_{s}$ in (46) equaling zero. If $\tau_{s}^{*}$ satisfies the inequality (45), $\tau_{s}^{*}$ is the optimal value of $\tau_{s}$ that maximizes the throughput $\lambda_{r}$. Otherwise, the lower bound of $\tau_{s}$ in (45) is the optimal value of $\tau_{s}$ due to the monotonicity analysis in (46)–(49).

Therefore, for the CRN with n=1, we have proved the existence of the optimal duration of the sensing period $\tau_{s}^{*}$ and provided an approach to obtain the explicit value.

## 5. Performance Analysis of the CRN with a Given Transmission Period

In this section, we tackle the trade-off between the duration of the sensing period and that of the reporting period for the throughput optimization and start our analysis by formulating the throughput and the collision probability.

Based on the definition of the throughput in (1), the throughput of the CRN with a given transmission period $\tau_{t}$, denoted by $\lambda_{t}$, is given by

(50) λt=πfτt∑x=knnx1−pf(τs)xpf(τs)n−x=πfτt(1−∑x=0k−1nx1−pf(τs)xpf(τs)n−x).

The collision probability of the CRN with a given transmission period, denoted by $P_{t}$, is given by

(51) Pt=πo∑x=knnx1−pd(τs)xpd(τs)n−x=πo(1−∑x=0k−1nx1−pd(τs)xpd(τs)n−x).

With respect to the given transmission period in this section, the mathematical relationship between $\tau_{s}$ and *n* can be formulated as

(52) $$\tau_{s}+n\tau_{c}=1-\tau_{t}.$$

### 5.1. Throughput Analysis

Notice that both $p_{f}\left(\tau_{s}\right)$ and (n−x) in (50) could be viewed as functions of *n*, so we derive partial derivatives of positive functions f(y) and g(y) as follows.

(53) $$\frac{\partial f{\left(y\right)}^{g(y)}}{\partial y}\;=\;f{\left(y\right)}^{g(y)}\;\left(\ln f(y)\frac{\partial g(y)}{\partial y}\;+\;\frac{g(y)}{f(y)}\;\cdot \;\frac{\partial f(y)}{\partial y}\right).$$

To tackle the trade-off between $\tau_{s}$ and $n\tau_{c}$, we differentiate $\lambda_{t}$ with respect to *n* as

(54) 1πfτt∂λt∂n=−∑x=0k−1∂nx∂n1−pf(τs)xpf(τs)n−x−∂pf(τs)∂n×∑x=0k−1nx1−pf(τs)xpf(τs)n−xn−xpf(τs)−x1−pf(τs)−lnpf(τs)∑x=0k−1nx1−pf(τs)xpf(τs)n−x≈−∑x=0k−1lnpf(τs)(n+1)n−x+1nx1−pf(τs)xpf(τs)n−x−∂pf(τs)∂n∑x=0k−1nx1−pf(τs)xpf(τs)n−xn−xpf(τs)−x1−pf(τs).

Then, we discuss the first term on the right-hand side of (54) as follows.

(55) $$\begin{array}{cc} \ln\left(\frac{p_{f}\left(\tau_{s}\right)\left(n+1\right)}{n-x+1}\right)<0 & \Rightarrow \frac{p_{f}\left(\tau_{s}\right)\left(n+1\right)}{n-x+1}<1\\ \; & \Rightarrow x<\left(1-p_{f}\left(\tau_{s}\right)\right)\left(n+1\right). \end{array}$$

As *x* is a non-negative integer in the closed interval [0,k−1], we obtain the sufficient condition that the first term on the right-hand side of (54) is positive as

(56) $$k<\left(1-p_{f}\left(\tau_{s}\right)\right)\left(n+1\right)+1.$$

Moreover, based on (28) and (52), we have

(57) $$\frac{\partial p_{f}\left(\tau_{s}\right)}{\partial n}=-\tau_{c}\frac{\partial p_{f}\left(\tau_{s}\right)}{\partial \tau_{s}}>0.$$

We observe from (54) and (57) that $\frac{\partial p_{f}\left(\tau_{s}\right)}{\partial n}>0$, nx>0, $p_{f}\left(\tau_{s}\right)>0$, and $1-p_{f}\left(\tau_{s}\right)>0$ hold; thus, the sufficient condition that the second term on the right-hand side of (54) is negative could be represented as

(58) $$\begin{array}{cc} \frac{n-x}{p_{f}\left(\tau_{s}\right)}-\frac{x}{1-p_{f}\left(\tau_{s}\right)}>0 & \Rightarrow x<(1-p_{f}\left(\tau_{s}\right))n. \end{array}$$

As *x* is a non-negative integer in the closed interval [0,k−1], we simplify the sufficient condition as

(59) $$k<(1-p_{f}\left(\tau_{s}\right))n+1.$$

We infer from (56) and (59) that the upper bound in (56) and that in (59) are approximately equal. Thus, we neglect the difference in the upper bounds to present the following observations concisely. As $\frac{\partial p_{f}\left(\tau_{s}\right)}{\partial \tau_{s}}$ in (28)–(30) is negative, we summarize the observations from (54)–(59) as follows.

- When $k\in (\left(1-p_{f}\left(\tau_{s}\right)\right)n+1,n]$, we deduce from (28)–(30) and (54) that the second term is dominant when $\tau_{s}$ tends to 0. Otherwise, the monotonicity of $\lambda_{t}$ with respect to the number of mini-slots *n* depends on $c_{1}$ and *k*. Compared with the second item as follows, the throughput $\lambda_{t}$ is more likely to increase with *n*.
- When $k\in [1,\left(1-p_{f}\left(\tau_{s}\right)\right)n+1]$, the first term on the right-hand side of (54) is positive, while the second term on the right-hand side of (54) is negative. We deduce from (28)–(30) and (57) that the second term is dominant when $\tau_{s}$ tends to 0. Thus, the throughput $\lambda_{t}$ decreases with *n* when $\tau_{s}$ tends to 0.

Due to the value interval of $p_{f}\left(\tau_{s}\right)$ in (42), the value interval of *k* in the first item occupies the majority part of the interval [1,n]. Moreover, we infer from (54) and (58) that, with the increase of *k*, the throughput $\lambda_{t}$ is more likely to increase with $n\tau_{c}$.

### 5.2. Collision Analysis

To analyze the monotonicity of the collision probability, we differentiate $P_{t}$ with respect to *n* as

(60) 1πo∂Pt∂n=−∑x=0k−1∂nx∂n1−pd(τs)xpd(τs)n−x−∂pd(τs)∂n×∑x=0k−1nx1−pd(τs)xpd(τs)n−xn−xpd(τs)−x1−pd(τs)−lnpd(τs)∑x=0k−1nx1−pd(τs)xpd(τs)n−x≈−∑x=0k−1lnpd(τs)(n+1)n−x+1nx1−pd(τs)xpd(τs)n−x−∂pd(τs)∂n∑x=0k−1nx1−pd(τs)xpd(τs)n−xn−xpd(τs)−x1−pd(τs).

For the first term on the right-hand side of (60), we have

(61) $$\begin{array}{cc} \ln\left(\frac{p_{d}\left(\tau_{s}\right)\left(n+1\right)}{n-x+1}\right)<0 & \Rightarrow \frac{p_{d}\left(\tau_{s}\right)\left(n+1\right)}{n-x+1}<1\\ \; & \Rightarrow x<\left(1-p_{d}\left(\tau_{s}\right)\right)\left(n+1\right). \end{array}$$

Since *x* is a non-negative integer in the closed interval [0,k−1], and nx>0, $1-p_{d}\left(\tau_{s}\right)>0$, and $p_{d}\left(\tau_{s}\right)>0$ hold, we obtain the sufficient condition that the first term on the right-hand side of (60) is positive as

(62) $$k<\left(1-p_{d}\left(\tau_{s}\right)\right)\left(n+1\right)+1.$$

Moreover, based on (35) and (52), we have

(63) $$\frac{\partial p_{d}\left(\tau_{s}\right)}{\partial n}=-\tau_{c}\frac{\partial p_{d}\left(\tau_{s}\right)}{\partial \tau_{s}}<0.$$

Then, we discuss the second term on the right-hand side of (60) as

(64) $$\begin{array}{cc} \frac{n-x}{p_{d}\left(\tau_{s}\right)}-\frac{x}{1-p_{d}\left(\tau_{s}\right)}>0 & \Rightarrow x<(1-p_{d}\left(\tau_{s}\right))n. \end{array}$$

Similarly, we obtain the sufficient condition that the second term on the right-hand side of (60) is positive as

(65) $$\begin{array}{c} k<(1-p_{d}\left(\tau_{s}\right))n+1. \end{array}$$

Therefore, when $k\in [\left(1-p_{d}\left(\tau_{s}\right)\right)\left(n+1\right)+1,n]$, the right-hand side of (60) depends on $c_{2}$ and *k*. When $k\in [\left(1-p_{d}\left(\tau_{s}\right)\right)n+1,\left(1-p_{d}\left(\tau_{s}\right)\right)\left(n+1\right)+1)$, the analysis of this case is similar to that of the third case as follows. Due to the limited value interval, this case could be neglected. When $k\in [1,\left(1-p_{d}\left(\tau_{s}\right)\right)n+1)$, both the first and second terms on the right-hand side of (60) are positive; thus, the collision probability $P_{t}$ increases with $n\tau_{c}$. Moreover, we infer from (62) and (65) that, with the increase of *k*, $P_{t}$ is more likely to decrease with $n\tau_{c}$.

### 5.3. Throughput Optimization

Based on the monotonicity analysis in Section 5.1 and Section 5.2, we formulate the throughput optimization problem as

(66) $$\begin{array}{cc} \; & \max_{n}\;\lambda_{t} \end{array}$$

(67) $$\begin{array}{cc} \; & s.t.\;P_{t}\leq P_{c}. \end{array}$$

The variable of the throughput optimization in (66) is the number of mini-slots *n*. (66) aims to optimize the time trade-off between the sensing period and the number of mini-slots in the reporting period. Based on the value interval of *k* discussed in Section 5.1 and Section 5.2, we summarize the throughput optimization as follows.

- When $k\in [\left(1-p_{f}\left(\tau_{s}\right)\right)\left(n+1\right)+1,n]$, the monotonicities of the throughput $\lambda_{t}$ and the collision probability $P_{t}$ depend on $c_{1}$, $c_{2}$, and *k*.
- When $k\in [\left(1-p_{d}\left(\tau_{s}\right)\right)\left(n+1\right)+1,\left(1-p_{f}\left(\tau_{s}\right)\right)\left(n+1\right)+1]$, the throughput $\lambda_{t}$ decreases with the number of mini-slots *n* when $\tau_{s}$ tends to 0. Comparing it with the third item, the collision probability $P_{t}$ is less likely to increase with the duration of the reporting period $n\tau_{c}$ due to the larger value of *k*. The optimal *n* depends on $c_{1}$, $c_{2}$ and *k*.
- When $k\in [1,\left(1-p_{d}\left(\tau_{s}\right)\right)\left(n+1\right)+1]$, the collision probability $P_{t}$ increases with the duration of the reporting period $n\tau_{c}$, and the maximum permissible collision probability $P_{c}$ provides an upper bound of *n* in the interval $\left(0,\frac{1-\tau_{t}}{\tau_{c}}\right]$. As $\frac{\partial \lambda_{t}}{\partial n}$ turns from negative to positive with the increase of $\tau_{s}$, the optimal $\tau_{s}$ depends on the upper bound of *n*.

## 6. Numerical Results

In this section, we evaluate the throughput and collision performances of the CRN with CSS. The numerical results consist of three parts, which correspond to the theoretical results in Section 3, Section 4 and Section 5, respectively. The impact of the “*k*-out-of-*n*” fusion rule on the throughput and collision performances is also presented for comparison. The parameters are set as follows unless otherwise specified. Without loss of generality, the length of a time slot is normalized as a unit time. The probability that the licensed spectrum is occupied is set to $\pi_{o}=0.2$, and the probability that the licensed spectrum is free is set to $\pi_{f}=0.8$. In accordance with the value intervals of $p_{d}\left(\tau_{s}\right)$ and $p_{f}\left(\tau_{s}\right)$ in Section 2.2, the parameter in $p_{f}\left(\tau_{s}\right)$ is set to $c_{1}=2$, and the parameter in $p_{d}\left(\tau_{s}\right)$ is set to $c_{2}=-2$.

**Figure 3:**
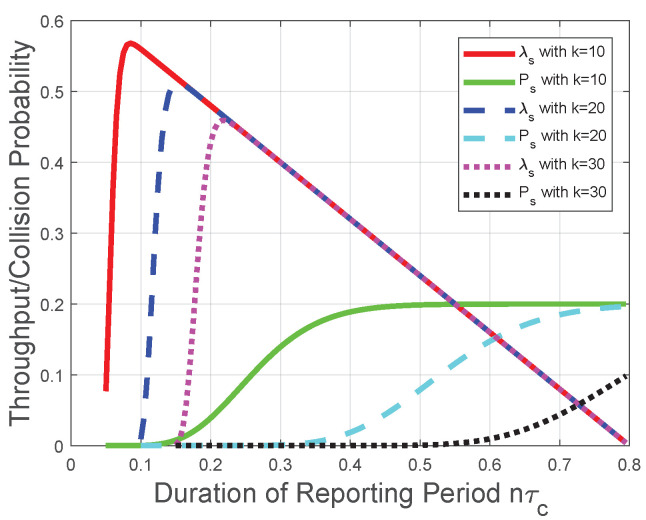
Throughput and collision performances of the CRN with a given sensing period.

Corresponding to the theoretical analysis in Section 3, Figure 3 plots the throughput $\lambda_{s}$ and the collision probability $P_{s}$ of the CRN with a given sensing period. We observe from Figure 3 that the throughput $\lambda_{s}$ increases with the number of mini-slots *n* when (17) holds; otherwise, the throughput $\lambda_{s}$ decreases with the number of mini-slots *n*. Thus, there is an optimal number of mini-slots for the throughput $\lambda_{s}$. When the number of mini-slots is larger than the optimal number of mini-slots, the throughput $\lambda_{s}$ decreases almost linearly due to the decrease of the duration of the transmission period $\tau_{t}$. Namely, $n\tau_{c}+\tau_{t}$ could be viewed as a constant in this scenario, and $\tau_{t}$ is represented as a multiplicator in the throughput formula ($1-\tau_{s}-n\tau_{c}$ in (8)). Moreover, with the increase of *k*, we observe from Figure 3 that the throughput $\lambda_{s}$ has a larger value interval where $\lambda_{s}$ increases with *n*, which is in accordance with the impact of the “*k*-out-of-*n*” fusion rule in Section 3.1. The reason behind this observation is that, based on the definition of the “*k*-out-of-*n*” fusion rule and the binomial coefficient in $\lambda_{s}$ (8), the FC has a higher probability to determine that the licensed spectrum is free when *n* increases. However, with the increase of *n*, the increase in the probability that FC determines the licensed spectrum is free could not compensate for the decrease of the transmission time in $\lambda_{s}$ (8); thus, the throughput $\lambda_{s}$ decreases with the number of mini-slots *n* when *n* is larger than the optimal value. Notice that this result is also in accordance with [16]. We also observe from Figure 3 that the collision probability $P_{s}$ increases with the number of mini-slots *n*; thus, $P_{s}$ provides an upper bound of *n* in the throughput optimization. This observation is in accordance with the collision analysis in Section 3.2 and the throughput optimization in Section 3.3. The reason is similar as that for the throughput $\lambda_{s}$. Based on the definition of the “*k*-out-of-*n*” fusion rule, the FC has a higher probability to determine the licensed spectrum is free when *n* increases. Therefore, the increase of *n* leads to more opportunities of spectrum access and more collisions with the primary packet transmission.

**Figure 4:**
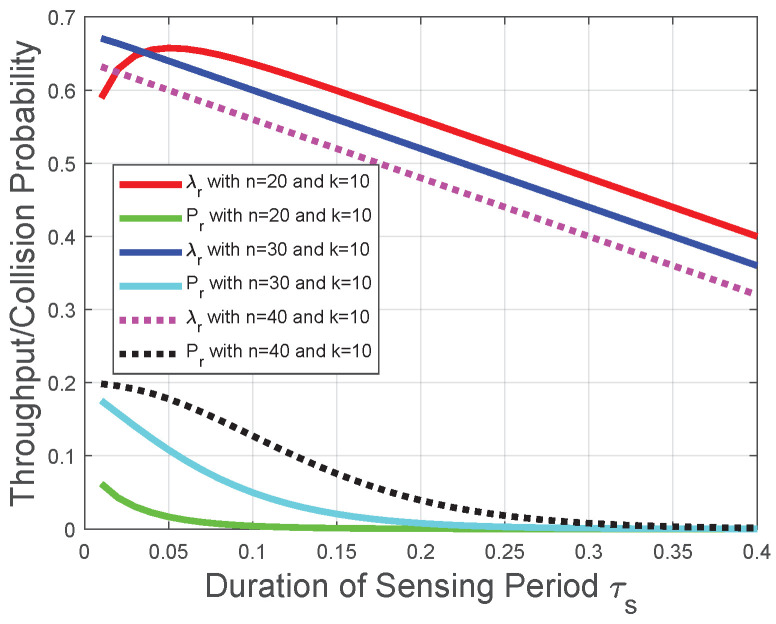
Throughput and collision performances of the CRN with a given reporting period and *k* in the “*k*-out-of-*n*” fusion rule.

**Figure 5:**
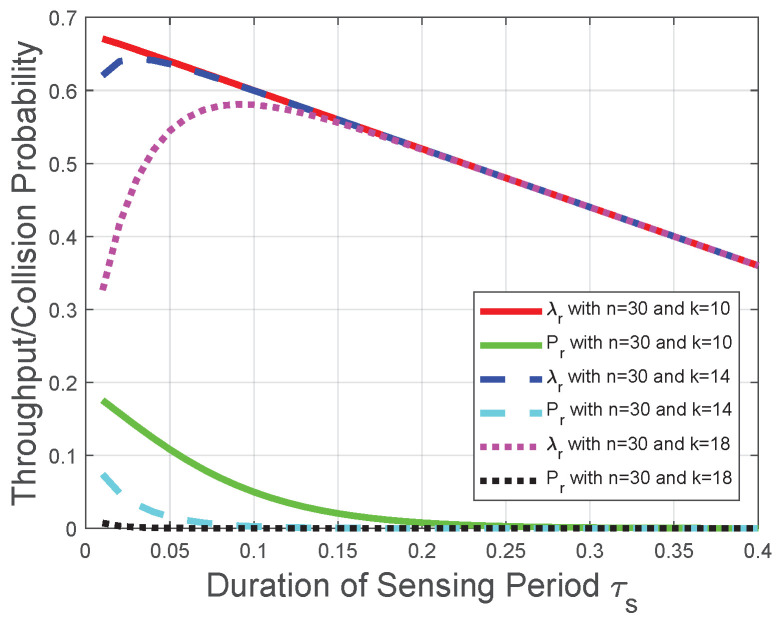
Throughput and collision performances of the CRN with a given reporting period and *n* in the “*k*-out-of-*n*” fusion rule.

**Figure 6:**
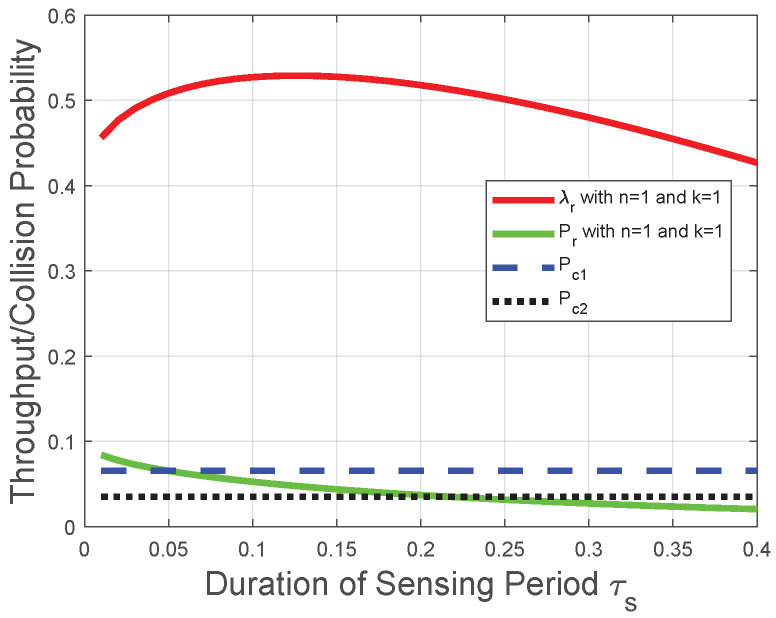
Throughput and collision performances of the CRN with a given reporting period, n=1, and k=1.

Corresponding to the theoretical analysis in Section 4.1, Section 4.2, Section 4.3, Figure 4 and Figure 5 plot the throughput $\lambda_{r}$ and the collision probability $P_{r}$ of the CRN with a given reporting period. In Figure 4, Figure 5 and Figure 6, we tackle the trade-off between the duration of the sensing period $\tau_{s}$ and the duration of the transmission period $\tau_{t}$ for the optimization problem in the CRN with a given number of mini-slots *n* in the reporting period and adopt $\tau_{s}$ as the variable on the horizontal axis to plot the performances of the throughput and the collision probability. We observe that the throughput $\lambda_{r}$ increases with the duration of the sensing period $\tau_{s}$ when $\tau_{s}$ tends to 0, otherwise the throughput $\lambda_{r}$ decreases with the duration of the sensing period $\tau_{s}$. This observation is in accordance with the throughput analysis in Section 4.1. The reason behind this observation is that, based on the throughput $\lambda_{r}$ in (25), the increase of $\tau_{s}$ leads to the decrease of the transmission time $\left(1-\tau_{s}-n\tau_{c}\right)$ and the increase of the probability, the FC determines the licensed spectrum is free. However, with the increase of $\tau_{s}$, the increase in the probability that FC determines the licensed spectrum is free could not compensate for the reduction of the transmission time in the throughput $\lambda_{r}$ (25). Thus, there is an optimal duration of the sensing period for the throughput $\lambda_{r}$, and the throughput $\lambda_{r}$ decreases with $\tau_{s}$ when the duration of the sensing period $\tau_{s}$ is larger than the optimal duration of the sensing period. The throughput $\lambda_{r}$ decreases almost linearly due to the decrease of the duration of the transmission period in (25). Figure 4 indicates that, with the decrease of *n*, the throughput $\lambda_{r}$ is more likely to increase with $\tau_{s}$. Figure 5 indicates that, with the increase of *k*, the throughput $\lambda_{r}$ is more likely to increase with $\tau_{s}$. These observations are also in accordance with the throughput analysis and the impact of the “*k*-out-of-*n*” fusion rule in Section 4.1. We also observe from Figure 4 and Figure 5 that the collision probability $P_{r}$ decreases with the duration of the sensing period $\tau_{s}$; thus, $P_{r}$ provides a lower bound of $\tau_{s}$ in the throughput optimization, which is in accordance with the collision analysis in Section 4.2 and the throughput optimization in Section 4.3. The reason behind this observation is that, based on (35), the probability of detection $p_{d}\left(\tau_{s}\right)$ increases with $\tau_{s}$. Based on the collision probability $P_{r}$ in (26), the FC has a lower probability to determine the licensed spectrum is free when $\tau_{s}$ increases. Therefore, the increase of $\tau_{s}$ leads to fewer opportunities of spectrum access and fewer collisions with primary-packet transmission.

Corresponding to the theoretical analysis in Section 4.4, Figure 6 plots the throughput $\lambda_{r}{|}_{n=1}$ and collision probability $P_{r}{|}_{n=1}$ versus the duration of the sensing period. We observe that $P_{r}{|}_{n=1}$ decreases with the duration of the sensing period $\tau_{s}$; thus, $P_{r}{|}_{n=1}$, and the maximum permissible collision probability $P_{c}$ provides a lower bound of $\tau_{s}$ in the throughput optimization. As for the case of the maximum permissible collision probability with the value $P_{c2}$, the throughput $\lambda_{r}{|}_{n=1}$ decreases with the duration of the sensing period $\tau_{s}$ under the lower bound of $\tau_{s}$, and this lower bound achieves the maximal throughput. As for the case with value $P_{c1}$, the throughput $\lambda_{r}{|}_{n=1}$ first increases with $\tau_{s}$ when it is relatively small and decreases with $\tau_{s}$ when it is relatively large; thus, the global optimal throughput could be achieved as shown in Figure 6. The above observations are in accordance with the performance analysis in Section 4.4. Moreover, we infer from Figure 3, Figure 4, Figure 5 and Figure 6 that when the duration of the sensing period $\tau_{s}$ is relatively large (does not tend to 0), the duration of the transmission period $\tau_{t}$ plays a much more important role than that of the reporting period or sensing period. The reason is that, due to the role of the transmission time as a multiplicator in the throughput Formulas (8) and (25), the throughput increases almost linearly with $\tau_{t}$.

**Figure 7:**
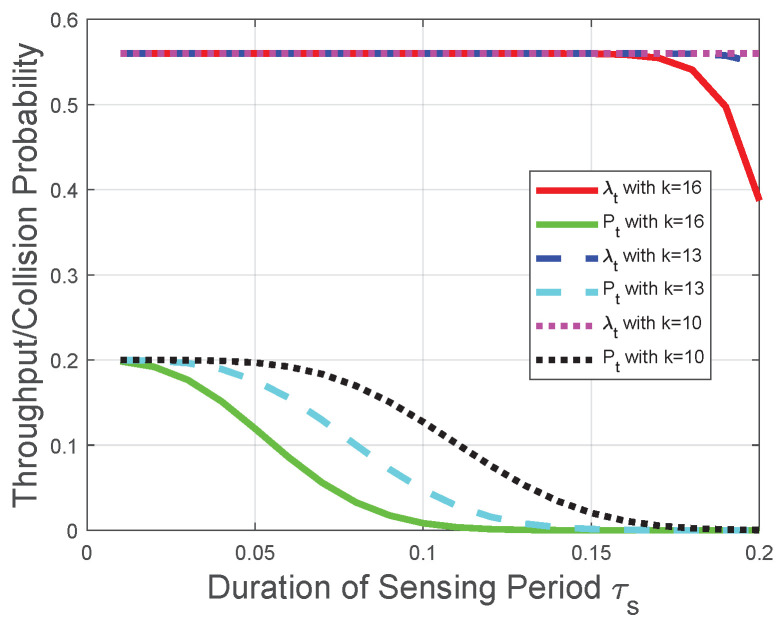
Throughput and collision performances of the CRN with a given transmission period when $\tau_{s}$ is relatively large.

**Figure 8:**
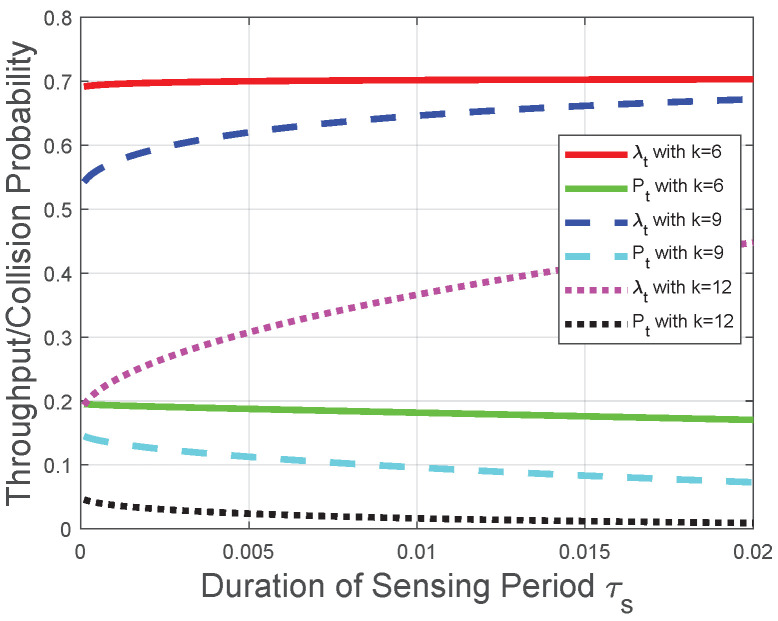
Throughput and collision performances of the CRN with a given transmission period when $\tau_{s}$ tends to 0.

Corresponding to the theoretical analysis in Section 5, Figure 7 and Figure 8 plot the throughput $\lambda_{t}$ and the collision probability $P_{t}$ of the CRN with a given transmission period. In Figure 7 and Figure 8, we tackle the trade-off between the duration of the sensing period $\tau_{s}$ and the number of mini-slots *n* in the reporting period for the optimization problem in the CRN with a given duration of the transmission period $\tau_{t}$ and adopt $\tau_{s}$ as the variable on the horizontal axis to plot the performances of the throughput and the collision probability. We observe that the throughput $\lambda_{t}$ increases with the duration of the sensing period $\tau_{s}$ when $\tau_{s}$ tends to 0; otherwise, the throughput $\lambda_{t}$ decreases with $\tau_{s}$. In the CRN with a given duration of the transmission period $\tau_{t}$, $\tau_{s}+n\tau_{c}$ could be viewed as a constant, and this observation is in accordance with the throughput analysis in Section 5.1. Namely, according to the given transmission period, the throughput $\lambda_{t}$ increasing with $\tau_{s}$ is equivalent to the throughput $\lambda_{t}$ decreasing with $n\tau_{c}$, and the observation from Figure 8 that the throughput $\lambda_{t}$ increases with $\tau_{s}$ when $\tau_{s}$ tends to 0 is also equivalent to the theoretical result in Section 5.1. The reason behind this observation is that, based on the definition of the “*k*-out-of-*n*” fusion rule and the binomial coefficient in $\lambda_{t}$ (50), the FC has a higher probability to determine the licensed spectrum is free when *n* or $\tau_{s}$ increases. As $\tau_{s}+n\tau_{c}$ could be viewed as a constant in this CRN scenario and $\tau_{c}$ is considered as a constant, the impact of $\tau_{s}$ on the throughput $\lambda_{t}$ is higher than that of *n* when $\tau_{s}$ tends to 0. The impact relationship between $\tau_{s}$ and $\lambda_{t}$ is due to the monotonicity of Q(·) in (2) and the binomial coefficient in $\lambda_{t}$ (50). We also observe from Figure 7 and Figure 8 that the collision probability $P_{t}$ decreases with $\tau_{s}$, which is in accordance with theoretical result in Section 5.2. Namely, the FC is less likely to determine that the licensed spectrum is free with the increase of $\tau_{s}$ (or equivalently, the decrease of *n* in this CRN scenario). Similarly, Figure 7 indicates that, with the increase of *k*, the throughput $\lambda_{t}$ is more likely to decrease with $\tau_{s}$, which is in accordance with the throughput analysis with the fusion rule in Section 5.1. The reason behind this indication is that, with the increase of *k*, the FC is less likely to determine the licensed spectrum is free according to the received *n* local sensing results. The increase of $\tau_{s}$ is equivalent to the decrease of *n* in this CRN scenario. Therefore, based on the binomial coefficient in (50), with the increase of *k*, the throughput $\lambda_{t}$ is more likely to decrease with $\tau_{s}$.

Moreover, we observe from Figure 3, Figure 4, Figure 5, Figure 6, Figure 7 and Figure 8 that both the throughput and the collision probability decrease with the parameter *k* in the “*k*-out-of-*n*” fusion rule, and this observation is in accordance with the definition of the “*k*-out-of-*n*” fusion rule. The reason behind this observation is that, with the increase of *k*, the FC is less likely to determine the licensed spectrum is free according to the received *n* local sensing results, resulting in a smaller opportunity of secondary-packet transmission and a smaller probability of collision.

## 7. Conclusions

In this paper, we have studied the throughput and collision performances of the cooperative CRN, where the crucial impact of the reporting design and that of the “*k*-out-of-*n*” fusion rule with a varying number of local sensing results are explored. To tackle the time trade-off among sensing, reporting, and transmission, theoretical evaluations and optimizations are performed in the CRNs with a given duration of the sensing/reporting/transmission period, respectively.

We have formulated the throughput and collision probability in the three cases of the CRN. In each case, the monotonicity analysis of the collision constraint and the maximum permissible probability provides an upper bound of *n* (Section 3.2 and Section 5.2) or a lower bound of $\tau_{s}$ (Section 4.2) for throughput maximization in the specific value intervals of sensing parameters. The monotonicity analysis of the throughput with the aforementioned upper/lower bounds provide approaches to the maximal throughput while satisfying the collision constraint. In the other value intervals of sensing parameters, the maximal throughput could be obtained with specified values of sensing and fusion parameters. The derived theoretical results are validated with numerical studies, especially analyzing the effect of the fusion parameters. The derived analytical results can be used to design a cooperative CRN with the required throughput and collision performances. Future works include more complex scenarios, such as the presence of malicious users that may make inappropriate use of the spectrum. 

## Data Availability

Not applicable.
